# Machine learning-based diagnostic model for preoperative differentiation between xanthogranulomatous cholecystitis and gallbladder carcinoma: a multicenter retrospective cohort study

**DOI:** 10.3389/fonc.2024.1355927

**Published:** 2024-02-27

**Authors:** Tianwei Fu, Yating Bao, Zhihan Zhong, Zhenyu Gao, Taiwei Ye, Chengwu Zhang, Huang Jing, Zunqiang Xiao

**Affiliations:** ^1^ General Surgery, Cancer Center, Department of Hepatobiliary & Pancreatic Surgery and Minimally Invasive Surgery, Zhejiang Provincial People's Hospital (Affiliated People's Hospital), Hangzhou Medical College, Hangzhou, Zhejiang, China; ^2^ Department of Hepatopancreatobiliary Surgery, The Affiliated Lihuili Hospital of Ningbo University, Ningbo University, Ningbo, Zhejiang, China

**Keywords:** xanthogranulomatous cholecystitis, gallbladder carcinoma, diagnostic nomogram, machine learning, preoperative imaging

## Abstract

**Background:**

Xanthogranulomatous cholecystitis (XGC) and gallbladder carcinoma (GBC) share similar imaging and serological profiles, posing significant challenges in accurate preoperative diagnosis. This study aimed to identify reliable indicators and develop a predictive model to differentiate between XGC and GBC.

**Methods:**

This retrospective study involved 436 patients from Zhejiang Provincial People’s Hospital and The Affiliated Lihuili Hospital of Ningbo University. Comprehensive preoperative imaging, including ultrasound, Computed Tomography (CT), Magnetic Resonance Imaging (MRI), and blood tests, were analyzed. Machine learning (Random Forest method) was employed for variable selection, and a multivariate logistic regression analysis was used to construct a nomogram for predicting GBC. Statistical analyses were performed using SPSS and RStudio software.

**Results:**

The study identified gender, Murphy’s sign, absolute neutrophil count, glutamyl transpeptidase level, carcinoembryonic antigen level, and comprehensive imaging diagnosis as potential risk factors for GBC. A nomogram incorporating these factors demonstrated high predictive accuracy for GBC, outperforming individual or combined traditional diagnostic methods. External validation of the nomogram showed consistent results.

**Conclusion:**

The study successfully developed a predictive nomogram for distinguishing GBC from XGC with high accuracy. This model, integrating multiple clinical and imaging indicators, offers a valuable tool for clinicians in making informed diagnostic decisions. The findings advocate for the use of comprehensive preoperative evaluations combined with advanced analytical tools to improve diagnostic accuracy in complex medical conditions.

## Introduction

Gallbladder diseases are frequently encountered in the clinical setting and comprise gallbladder malignant carcinoma and xanthogranulomatous cholecystitis (XGC). It is vital to correctly diagnose these two diseases, given their contrasting treatment options. Xanthogranulomatous cholecystitis is a special pathological type of chronic cholecystitis (1–3). In clinical practice, it is challenging to distinguish xanthogranulomatous cholecystitis from gallbladder carcinoma (GBC) via preoperative examinations (4). The imaging features of XGC and GBC include gallbladder wall thickening, gallbladder wall enhancement, and invasion of surrounding tissues (5, 6). They also have similar clinical manifestations, such as abdominal pain, jaundice, weight loss, and loss of appetite. Xanthogranulomatous cholecystitis is usually treated by cholecystectomy. However, owing to the similarities in imaging results and a lack of specific serological biomarkers, xanthogranulomatous cholecystitis is often misdiagnosed. Indeed, the diagnosis can only be confirmed via pathological examination or fine-needle aspiration following cholecystectomy (7, 8). The latter is not a recommended diagnostic option according to current guidelines because of the potential risks of bile leakage, tumor dissemination, and sampling error. Therefore, there is an urgent need to develop a non-invasive method to pre-operatively distinguish between XGC and GBC.

Abdominal ultrasound, computed tomography, magnetic resonance imaging, and other imaging examinations are routine preoperative examinations performed for XGC and GBC patients. The thickening pattern of the gallbladder wall can be divided into two types, namely focal and diffuse (9). Although the pattern of wall thickening in gallbladder cancer differs from that in benign disease, it is less specific. Therefore, there are many cases of misdiagnosis in clinical work (10). In addition, XGC can coexist with GBC (11). Due to the comparable clinical manifestations and imaging features, it is difficult to differentiate between XGC and GBC in the clinical setting. Therefore, XGC is frequently misdiagnosed as GBC, leading to unnecessary radical cholecystectomy and significantly increasing the complexity of the surgical intervention and the incidence of intraoperative and postoperative complications, such as biliary fistula, surgical site infection, bleeding, and organ damage. Conversely, GBC may also be misdiagnosed as XGC, and incomplete preoperative evaluation leads to missing the optimal treatment window or unnecessary surgical treatment for GBC patients. At present, with the exception of postoperative pathological diagnosis, there is no satisfactory preoperative model to distinguish GBC from xanthogranulomatous cholecystitis. Therefore, accurate clinical diagnosis is essential for the ensuing treatments of XGC and GBC patients.

## Materials

**Figure 1:**
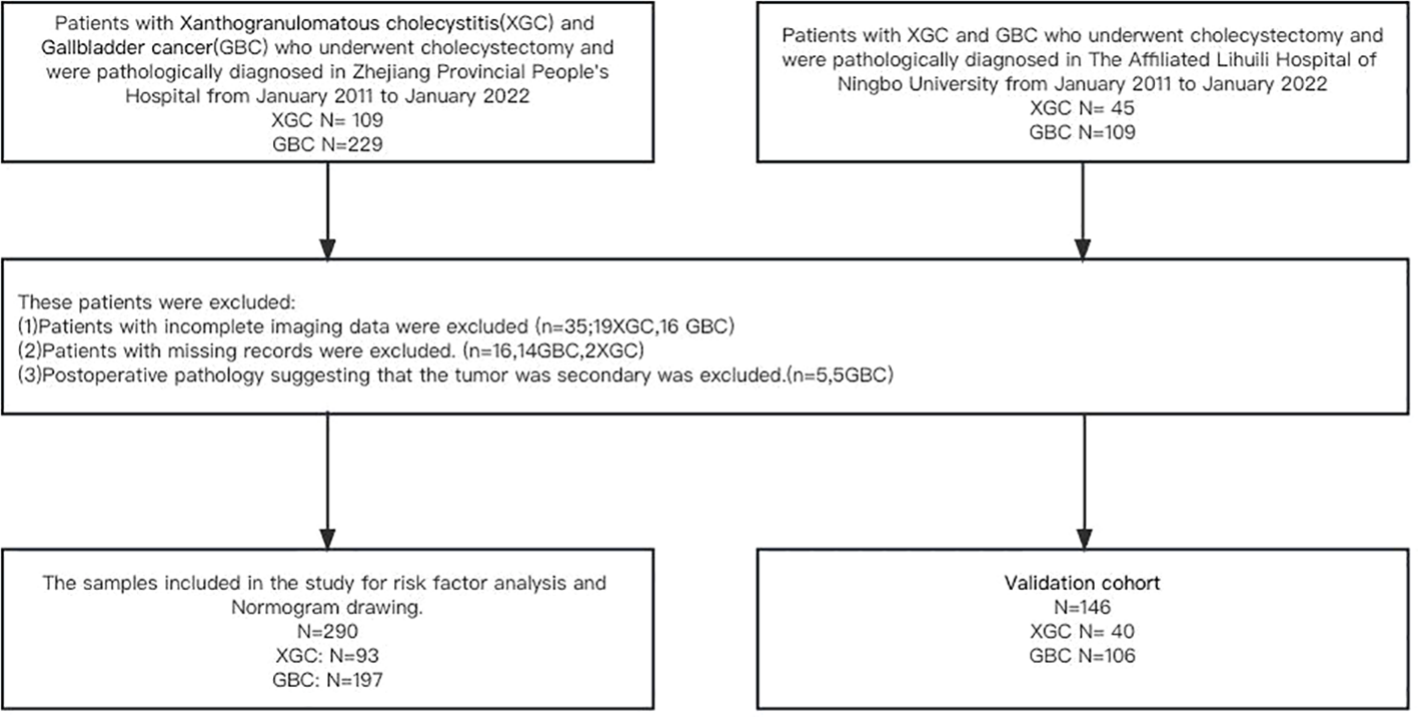
Flow diagram of the study design. A total of 290 patients with postoperative pathological diagnosis of XGC and GBC were included in the study of Zhejiang Provincial People’s Hospital, including 93 patients with XGC and 193 patients with GBC. The Affiliated Lihuili Hospital of Ningbo University was included in this study as a validation cohort.

The subjects were patients who underwent cholecystectomy and a histologically-confirmed diagnosis of XGC and GBC in Zhejiang Provincial People’s Hospital and The Affiliated Lihuili Hospital of Ningbo University from January 2011 to January 2022. The clinical data of 109 patients with XGC were retrospectively analyzed, and 16 patients with incomplete imaging data were excluded. Similarly, the clinical data of 229 patients with GBC were analyzed, among which 14 patients with missing data, 16 patients with incomplete imaging data, and 2 patients with secondary gallbladder malignancy were excluded. Finally, 93 patients with XGC and 197 patients with GBC were eligible to participate in the training cohort, and the patients were divided into the XGC and GBC groups. Similarly, 40 patients with XGC and 106 patients with GBC were enrolled in The Affiliated Lihuili Hospital of Ningbo University as the validation cohort. Details of the inclusion and exclusion criteria for patients in this study are illustrated in **Figure 1**.

The comprehensive preoperative imaging diagnosis was conducted through abdominal ultrasound, abdominal-enhanced computed tomography (CT), magnetic resonance cholangiopancreatography (MRCP), and abdominal-enhanced magnetic resonance (MRI). Due to the absence of retrospective imaging records, the abdominal ultrasound diagnosis relied solely on its report. The imaging data of both training and validation cohorts were reviewed by the same two experienced radiologists, who were blinded to the patient’s clinical, laboratory, or pathological details during re-evaluation. If their interpretations differed, a consensus was reached through discussion and mutual consensus. The Kappa-Cohen index was used to assess inter-observer agreement. Comprehensive preoperative imaging diagnosis represents the comprehensive evaluation of the patient by imaging, and the evaluation criteria are as follows: (1) When all imaging examinations (ultrasound, CT, and MRI) indicate benign gallbladder disease, the preoperative diagnosis was defined as “benign gallbladder disease”. (2) When the results of imaging examination in the diagnosis of benign and malignant gallbladder were inconsistent, the preoperative comprehensive diagnosis was “suspected GBC”. (3) The preoperative diagnosis was labeled as “GBC” if all imaging tests uniformly suggested malignancy.

All experiments were conducted in accordance with the Declaration of Helsinki. All procedures performed involving human participants in the study adhered to the ethical standards of the Institutional Review Board (IRB). Due to the study’s retrospective design, involving the use and analysis of past data, the IRB approved a waiver for the requirement of written informed consent.

## Methods

Continuous variables following a normal distribution were represented by mean ± standard deviation (SD), while those following a skewed distribution were expressed as quartile range (IQR) and median. For comparison, the Mann-Whitney U test was used. Frequency data were expressed as numbers and percentages and compared using the Chi-square test or Fisher’s exact test. The receiver-operating characteristic (ROC) curve analysis was used to assess the ability of each tumor marker and imaging method to differentiate xanthogranulomatous cholecystitis (XGC) from GBC (GBC).

The “Random Forest” method in machine learning was used for model variable selection. After the transformation of continuous variables into binary variables according to the best cutoff value, all variables were included in the multivariate logistic regression analysis according to their importance. Collinearity diagnosis was conducted for each variable included in the model. The OR and 95% CI of each independent risk factor in the model were calculated. A nomogram was subsequently constructed to predict the probability of GBC. The Hosmer-Lemesow test was used to evaluate the suitability of the nomogram (P > 0.05 indicating good fit), and correction curves were plotted to compare the relationship between the predicted probability and the actual probability. Sensitivity, specificity, positive predictive value (PPV), negative predictive value (NPV), and 95% CI were calculated for different risk cutoff points. The data set of The Affiliated Lihuili Hospital of Ningbo University was used for external validation of the nomogram. DeLong’s test was used to estimate the difference between the ROC curves of the training and validation cohorts.

All statistical analyses were performed using SPSS software (version 26.0.0.0) and RStudio software (RStudio 2022.07.2 + 576,© 2009-2022 RStudio, PBC). All reported levels of statistical significance were bilateral, and statistical significance was set to 0.05.

## Results

In this study, clinical data of 290 patients in Zhejiang Provincial People’s Hospital and 146 patients in The Affiliated Lihuili Hospital of Ningbo University was collected and analyzed. **Table 1** summarizes the demographic characteristics, clinical symptoms, and data on laboratory and imaging data for both the training and validation cohorts. Univariate analysis revealed that gender, right upper quadrant abdominal pain, vomiting, abdominal distention, Murphy’s sign, radiating pain, history of gallstones, absolute neutrophil count (N), prothrombin time (PT), comprehensive preoperative imaging diagnosis, as well as levels of albumin (ALB), GGT, alkaline phosphatase (ALK), direct bilirubin (DBIL), and CEA were potential risk factors for the differential diagnosis of XGC and GBC (all P < 0.05).

**Table 1:** Demographic and clinical characteristics of the study population.

Baselines variables	Training cohort				Validation cohort			
	Total (n = 290)	Xanthogranulomatous cholecystitis (n = 93)	Gallbladder carcinoma (n = 197)	P value	Total (n = 146)	Xanthogranulomatous cholecystitis (n =40)	Gallbladder carcinoma (n =106 )	P value
Gendar, female (%)	166 (57.2%)	34 (36.6%)	132 (67.0%)	< 0.001	77(52.7%)	14(35.0%)	63(59.4%)	0.010
Age(years),median (IQR)	64.90 ±7.0	63.75 ±14.3	65.44 ±10.6	0.314	66.85 ±10.91	63.80 ±13.73	68.00 ±9.45	0.080
BMI(kg/m2),mean (SD)	22.79 ± 3.19	23.26 ± 2.78	22.57 ± 3.36	0.066	23.54 ±3.12	24.40 ±3.94	23.21±2.70	0.039
Epigastric pain, n (%)	213 (73.4%)	88 (94.6%)	125 (63.5%)	< 0.001	83(56.8%)	28(70.0%)	55(51.9%)	0.061
Ventosity, n (%)	92 (31.7%)	42 (45.2%)	50 (25.4%)	0.001	46(31.5%)	15(37.5%)	31(29.2%)	0.452
Fever, n (%)	81 (27.9%)	31 (33.3%)	50 (25.4%)	0.205	35(24.0%)	11(27.5%)	24(22.6%)	0.542
Emesis, n (%)	75 (25.9%)	32 (34.4%)	43 (22.8%)	0.032	29(19.9%)	11(27.5%)	18(17.0%)	0.168
Jaudice, n (%)	58 (20.0%)	22 (24.7%)	36 (18.3%)	0.362	23(15.8%)	4(10.0%)	19(17.9%)	0.313
Murphy, n (%)	79 (27.2%)	57 (61.3%)	22 (11.2%)	< 0.001	23(15.8%)	16(42.5%)	6(5.7%)	< 0.001
Radiating pain, n (%)	82 (27.2%)	41 (44.1%)	41 (20.8%)	< 0.001	32(21.9%)	10(25.0%)	22(20.8%)	0.365
Weight loss, n (%)	23 (7.9%)	7 (7.5%)	16 (8.1%)	1	11(7.5%)	1(2.5%)	10(9.4%)	0.290
Abdominal surgery, n (%)	14 (4.8%)	3 (3.2%)	11 (5.6%)	0.561	9(6.2%)	4(10.0%)	5(4.7%)	0.258
Malignant individual, n (%)	30 (10.3%)	14 (15.1%)	16 (8.1%)	0.109	12(8.2%)	3(7.5%)	9(8.5%)	1
Personal gallstones history, n (%)	195 (67.6%)	82 (88.2%)	114 (57.9%)	< 0.001	91(62.3%)	33(82.5%)	58(54.7%)	0.002
Personal polyp history, n (%)	18 (6.2%)	2 (2.2%)	16 (8.1%)	0.082	4(2.7%)	1(2.5%)	3(2.8%)	1
L(*10^9/L), median (IQR)	1.48 (1.10, 1.88)	1.50 (1.10, 1.89)	1.44 (1.10, 1.88)	0.989	1.5(1.1, 1.9)	1.41 (1.04, 1.85)	1.50 (1.10, 1.90)	0.656
N(*10^9/L), median (IQR)	4.23 (3.07, 6.79)	5.95 (3.38, 9.03)	4.00(3.00, 5.52)	< 0.001	4.43(3.30, 6.25)	5.92(3.39, 7.48)	4.40(3.23, 5.70)	0.024
M(*10^9/L), median (IQR)	0.37 (0.30, 0.51)	0.40 (0.30, 0.60)	0.34 (0.29, 0.50)	0.008	0.50(0.40, 0.63)	0.44(0.30, 0.60)	0.50 (0.40, 0.70)	0.028
ALB(g/L), mean (SD)	38.13 ± 5.39	36.38 ± 5.18	38.97 ± 5.29	< 0.001	39.10±6.21	36.63±6.15	40.03± 5.99	0.003
ALT (U/L), median (IQR)	28 (14, 90)	39 (17, 95)	26(14,88)	0.068	26(17,74)	36(20, 116)	24(17,74)	0.439
AST(U/L), median (IQR)	28 (21, 69)	31 (20, 75)	27 (21, 68)	0.918	27(21,48)	31(21, 80)	26(20,46)	0.335
GGT(U/L), median (IQR)	60 (25, 261)	125 (41, 266)	40 (21, 253)	< 0.001	60(30,196)	126(35, 237)	51(24,167)	0.032
ALP(U/L),median (IQR)	114 (82, 223)	130 (96, 236)	105 (80, 212)	0.021	104(78,186)	114(83, 177)	98(75,208)	0.796
DBIL(umol/L), median (IQR)	3.6 (2.2, 12.3)	5.1 (2.5, 13.9)	3.2 (2.2, 9.7)	0.042	4.3 (2.7, 9.5)	4.1 (2.4, 9.7)	4.6(3.1,9.3)	0.114
IBIL(umol/L), median (IQR)	12.2 (8.8, 20.6)	12.4 (9.0, 23.4)	12.1 (8.8, 19.5)	0.612	8.9 (6.9, 13.7)	10.8 (8.0, 19.7)	8.5(6.4,11.9)	0.017
TBA(umol/L), median (IQR)	6.9 (3.7, 17.6)	7 .0(4.0, 15.6)	6.8 (3.5, 21.1)	0.970	6.0(3.3, 18.8)	5.8 (3.3, 13.9)	20.7(6.0,196.7)	0.729
PT(s), median (IQR)	11.8 (11.1, 12.5)	12.1 (11.4, 12.8)	11.7 (11.0, 12.3)	< 0.001	11.6(11.0, 12.2)	11.7 (11.3, 12.3)	11.5(10.9,12.1)	0.178
AFP(ug/L), median (IQR)	2.4 (1.8, 3.4)	2.1 (1.7, 3.3)	2.5 (1.9, 3.6)	0.079	2.5 (1.7, 4.0)	2.3 (1.7, 3.1)	2.6(1.7,4.5)	0.219
CEA(ug/L), median (IQR)	2.6 (1.6, 5.1)	2.0 (1.2, 2.9)	2.9 (1.8, 5.7)	< 0.001	1.8 (1.2, 5.2)	1.6 (1.1, 2.9)	1.9(1.2,6.8)	0.047
CA125(U/L), median (IQR)	16.8 (10.6, 33.0)	21.9 (11.5, 38.0)	16.6 (10.6, 30.3)	0.183	17.8(9.7, 42.1)	13.0(10.3, 30.5)	18.8(9.2,50.7)	0.146
CA19-9(U/L), median (IQR)	37.4 (13.0, 180.5)	54.2 (15.0, 194.0)	33.1 (12.5, 154.1)	0.150	49.4 (12.8, 316.7)	52.9 (12.5, 94.9)	49.1(13.4,461.9)	0.203
CA15-3(U/L), median (IQR)	11.0 (8.5, 16.7)	10.7 (8.1, 15.7)	11.5 (8.7, 17.1)	0.197	11.5 (7.3,15.6)	12.2 (7.0, 16.5)	11.1(7.5,15.4)	0.640
Comprehensive preoperative imaging diagnosis				< 0.001				< 0.001
Benign diseases of gallbladder	117 (40.3%)	77 (82.8%)	40 (20.3%)		55(37.7%)	33(82.5%)	17(19.8%)	
Suspected gallbladder carcinoma	68 (23.4%)	8 (8.6%)	60(30.5%)		48(32.9%)	3(7.5%)	42(42.5%)	
Gallbladder carcinoma	105 (36.2%)	8 (8.6%)	97 (49.2%)		43(29.5%)	4(10.0%)	47(37.3%)	

XGC, Xanthogranulomatous; GBC, Gallbladder carcinoma; L, lymphocyte; N, neutrophile granulocyte; M, monocyte; ALB, serum albumin; ALT, alanine aminotransferase; AST, aspartate aminotransferase; GGT, gamma-glutamyl transpeptidase; ALP, alkaline phosphatase; DBIL, direct bilirubin; IBIL, indirect bilirubin; TBA, total bile acid; PT, prothrombin time; AFP, alpha-fetal protein; CA 19-9, cancer antigen 19-9; CEA, carcinoembryonic antigen; CA 153, cancer antigen 153.

**Figure 2:**
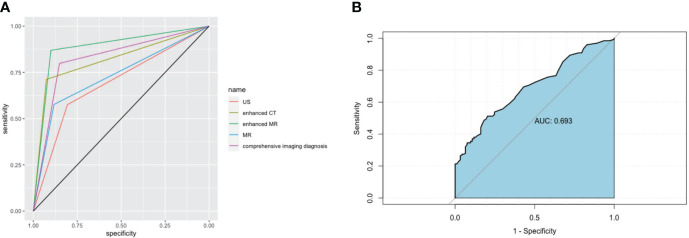
Imaging and receiver operating characteristic (ROC) curve analysis of serum markers in XGC and GBC patients prior to cholecystectomy. **(A)** The areas under the AUC curve were 0.691, 0.819, 0.885, 0.729, and 0.821, respectively, for ultrasonography, enhanced CT, enhanced MRI, plain MRI, and comprehensive imaging diagnosis. **(B)** Serum CEA. The critical value of serum CEA (μg/L) in differentiating XGC and GBC showed that the area under the AUC curve was 0.693.

The results of preoperative ultrasonography, enhanced CT, MRI, and enhanced MR of patients are summarized in **Table 2**. As displayed in **Figure 2**, contrast-enhanced CT and contrast-enhanced MRI had the highest discriminatory power, with AUCs (95% CI) of 0.819 (0.769–0.868) and 0.885 (0.764–1), respectively. Comprehensive preoperative imaging had an AUC (95% CI) of 0.821 (0.772–0.869). However, the AUC (95% CI) of unenhanced MRI was 0.729 (0.665–0.793), while that of the US was 0.692 (0.638–0.746). The inter-observer agreement between the two radiologists was good. The Kappa-Cohen index was 0.810 in the training cohort and 0.782 in the validation cohort.

**Table 2:** The simple frequency distribution of preoperative US, enhanced CT, MR, enhanced MR and comprehensive imaging diagnosis results in XGC and GBC groups was shown.

		XGC	GBC	Total
US	Benign gallbladder disease	73	79	152
	GBC	20	118	138
enhanced CT	Benign gallbladder disease	62	40	102
	GBC	5	99	104
MR	Benign gallbladder disease	45	47	92
	GBC	6	64	70
enhanced MR	Benign gallbladder disease	9	3	12
	GBC	1	20	21
comprehensive imaging diagnosis	Benign gallbladder disease	77	40	117
	GBC	16	157	173
	Total	93	197	290

**Figure 3:**
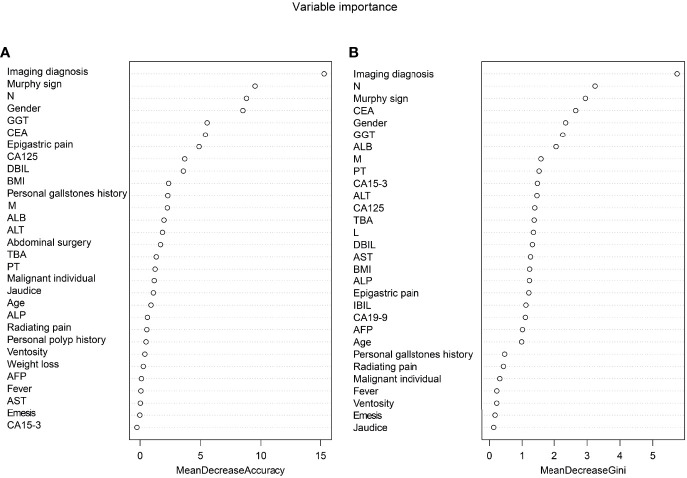
Ranking of input variables in the random forest model to predict GBC. **(A)** The mean decrease in accuracy. **(B)** Mean decrease Gini. Variables are listed from most important to least important based on the mean decrease in accuracy and mean reduction in the Gini coefficient.

The thirty-three characteristic variables in the study were included and ranked by importance using the random forest method (**Figure 3**). Finally, the top six variables with Mean decrease Gini (A larger value indicates a greater importance of the variable) were selected for inclusion in the model, and the continuous variables included N, GGT, and CEA. For the convenience of model construction and scoring, continuous variables were divided into binary variables according to the best cutoff value of ROC curve analysis (**Table 3**).

**Table 3:** Univariate analysis of the GGT, N and CEA as a categorical variable.

Laboratory test index	Total (n = 290)	Xanthogranulomatous cholecystitis (n = 93)	Gallbladder carcinoma (n = 197)	P value
GGT(U/L)				< 0.001
<29	83 (29%)	7 (8%)	76 (39%)	
≥29	207 (71%)	86 (92%)	121 (61%)	
N(*10^9/L)				< 0.001
<5.60	188 (67%)	38(41%)	150(76%)	
≥5.60	102 (33%)	55(59%)	47(24%)	
CEA(ug/L)				< 0.001
<3.2	170 (5%)	74(80%)	96(49%)	
≥3.2	120 (%)	19(20%)	101(51%)	

XGC, Xanthogranulomatous; GBC, Gallbladder carcinoma; N, neutrophile granulocyte; GGT, gamma-glutamyl transpeptidase; CEA, carcinoembryonic antigen.

### Serum CEA

The preoperative serum CEA levels of 93 patients in the XGC group and 197 patients in the GBC group were statistically analyzed. The median level of serum carcinoembryonic antigen in the GBC group was 2.9 µg/L (range: 0.2–244.9 µg/L), which was significantly higher than 2.0 µg/L in the XGC group (range: 0.4–6.9 µg/L) (P < 0.001). The ROC curve of serum carcinoembryonic antigen value for diagnosis was then drawn. The AUC value was found to be 0.693. According to the ROC curve analysis of CEA level, the optimal cutoff value of CEA was 3.2 ug/L when the Youden index was the highest. The sensitivity and specificity were 51.3% and 79.6%, respectively (**Figure 2B**). Subsequently, unifactor analysis was performed (**Table 3**). The difference between the two groups was statistically significant (P < 0.001).

Multiple Logistic regression analysis was conducted to identify independent predictors for differentiating XGC from GBC. Then, stepwise inclusion and exclusion methods were used to analyze the model, which finally yielded six independent predictors for the differentiation of XGC and GBC (**Table 4**). In the comprehensive preoperative imaging diagnosis, compared with “benign gallbladder disease,” the OR value of “GBC” was (OR = 17.45; 95% CI: 6.61–46.06), and the OR value of “suspected GBC” was (OR = 13.51; 95% CI: 4.87–37.44). Similarly, female gender (OR = 4.21; 95% CI: 1.91–9.32) and CEA ≥ 3.2μg/L (OR = 4.05; 95% CI: 1.31–12.46) were independent risk factors for the diagnosis of GBC. Contrastingly, a positive Murphy’s sign, GGT ≥ 29 U/L, and N ≥ 5.60 × 10^9^/L were associated with a lower risk of GBC, with OR values of 0.15 (95% CI: 0.07–0.34), 0.27 (95% CI: 0.09–0.82), and 0.41 (95% CI: 0.18–0.91), respectively.

**Table 4:** Multivariate Logistic regression analysis of risk factors for XGC and GBC.

Characteristic	Comparisons	UV OR(95% CI)	UV P	MV OR(95% CI)	MV P
Gendar, female (%)	Female vs. male	3.52(2.10-5.90)	<0.01	4.21(1.91-9.32)	<0.01
Murphy,yes	Yes vs. no	0.08(0.04-0.15)	<0.01	0.15(0.07-0.34)	<0.01
N≥5.60*10^9/L	Yes vs. no	0.22(0.13-0.37)	<0.01	0.41(0.18-0.91)	0.041
GGT≥29U/L	Yes vs. no	0.13(0.06-0.30)	<0.01	0.27(0.09-0.82)	0.004
CEA≥3.2 ug/L	Yes vs. no	4.10(2.30-7.29)	<0.01	4.05(1.31-12.46)	0.004
Comprehensive preoperative imaging diagnosis	Suspected gallbladder carcinoma vs. benign gallbladder diseases	14.43(6.29-33.13)	<0.01	13.51(4.87-37.44)	<0.01
	Gallbladder carcinoma vs. benign gallbladder diseases	23.34(10.32-52.78)	<0.01	17.45(6.61-46.06)	<0.01

XGC, Xanthogranulomatous ; GBC, Gallbladder carcinoma; N, neutrophile granulocyte; GGT, gamma-glutamyl transpeptidase; CEA, carcinoembryonic antigen.

**Figure 4:**
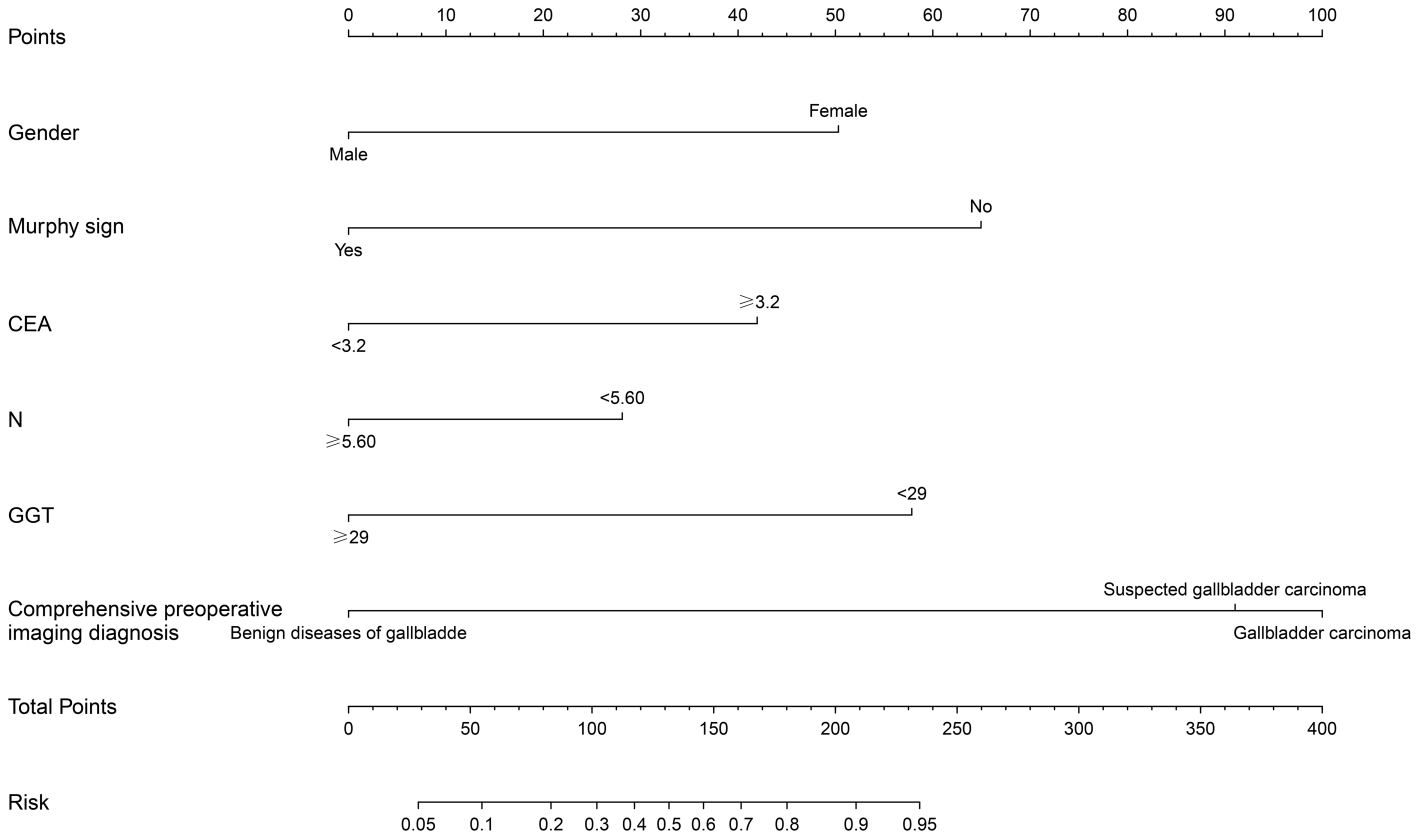
Construction of nomogram of xanthogranulomatous cholecystitis (XGC) and GBC (GBC) patients. The nomogram was established according to six possible independent predictors of cancer: gender, Murphy’s sign, CEA, neutrophils absolute value (N), glutamyl transpeptienzyme (GGT), and comprehensive preoperative imaging diagnosis. For each patient, the values of the six risk factors are represented as dots by projecting them onto the topmost line (point scale). Summing the six variables and projecting the total points’ value downward onto the bottom-most line can determine the probability of GBC.

**Figure 5:**
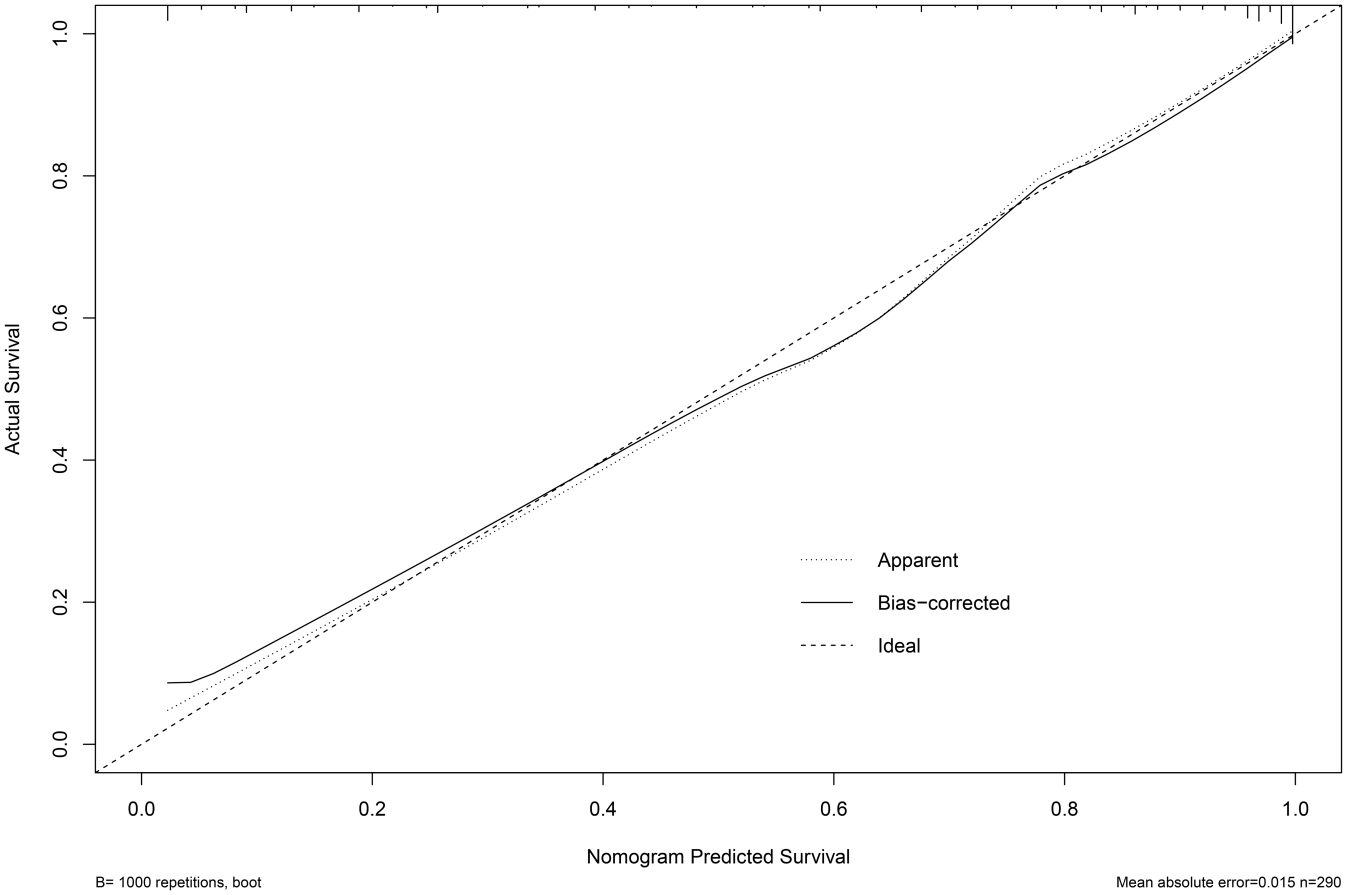
Calibration plot of Normogram for the probability of diagnosis of gallbladder malignancy.

**Figure 6:**
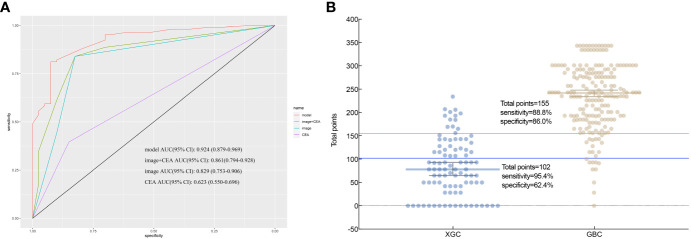
According to nomogram scores, the performance of the novel model (nomogram), image+CEA, image, and CEA in differentiating patients with xanthogranulomatous cholecystitis (XGC) and GBC (GBC) were compared. **(A)** Receiver operating characteristic (ROC) curve. The area under the ROC curve of the model, image+CEA, image, and CEA, was 0.936, 0.861, 0.821, and 0.654, respectively. It reflects the good discriminant ability of the nomogram to predict GBC. **(B)** Interactive dot diagram for the performance of the nomogram in differentiating XGC and GBC. X-axis: patients in XGC and GBC groups; Y-axis: the scale for total points of each XGC and GBC patient; each dot; each dot is a data point for the result of each patient. The horizontal blue line represents the optimal critical value that maximizes the sum of sensitivity and specificity.

Based on the multifactor model, the nomogram was constructed with the six independent risk factors (**Figure 4**). In the nomogram, each predictor was assigned a score according to its classification. A patient’s total score corresponded to the likelihood of GBC. The p-value of the Hosmer-Lemesshow test for this model was 0.700 (P > 0.05, good model fitting) (**Figure 5**). Subsequently, a nomogram calibration chart was developed to evaluate the predictive value of the model by curves of prediction probability and actual probability. The AUC of the nomogram was 0.936 (95% CI, 0.909–0.963), with accuracy, sensitivity, specificity, NPV, and PPV of 87.2%, 76.3%, 92.4%, 82.6%, and 89.2%, respectively. Moreover, the optimal cutoff value of the nomogram was 0.65, corresponding to 155 points, and the corresponding sensitivity and specificity were 88.8% and 86.0%, respectively. Furthermore, comparing the AUCs revealed that among the two commonly used combinations, the nomogram had the highest discriminant ability and outperformed that of the combination of radiographic diagnosis and CEA levels, which had an AUC of 0.861 (P < 0.01) (**Figure 6**). According to the American Joint Committee on Cancer (AJCC) GBC staging, the model characteristics of GBC patients in the training cohort are demonstrated in **Table 5**. As shown in **Table 6**, comparing the preoperative imaging examination and intraoperative frozen results of GBC patients with AJCC stage I and II, there was no statistically significant difference between them.

**Table 5:** Distribution of model characteristics in each AJCC stage of GBC patients.

Characteristic	AJCC stage I (n=37)	AJCC stage II (n=36)	AJCC stage III (n=78)	AJCC stage IV (n=46)	Total (n=197)
Gendar, female (%)	22(59.5%)	23(63.9%)	55(70.5%)	32(69.6%)	132(67.0%)
Murphy, no	34(91.9%)	33(91.7%)	71(91.0%)	38(82.6%)	175(88.8%)
N <5.60*10^9/L	29(78.4%)	29(80.6%)	67(85.9%)	25(54.3%)	150(76.1%)
GGT <29U/L	14(37.8%)	19(52.8%)	28(35.9%)	15(32.6%)	76(38.6%)
CEA ≥3.2 ug/L	12(32.4%)	17(47.2%)	45(57.7%)	27(58.7%)	101(51.3%)
Comprehensive preoperative imaging diagnosis					
Benign gallbladder diseases	9(24.4%)	13(36.2%)	13(16.7%)	5(10.9%)	40(20.3%)
Suspected gallbladder carcinoma	14(37.8%)	8(22.2%)	25(32.1%)	13(28.3%)	60(30.5%)
Gallbladder carcinoma	14(37.8%)	15(41.6%)	40(51.2%)	28(60.8%)	97(49.3%)
Total points	226(160,279)	234(184,291)	250(193,293)	243(181,279)	242(185,285)

AJCC, American Joint Committee on Cancer; GBC, Gallbladder carcinoma; N,neutrophile granulocyte; GGT, gamma-glutamyl transpeptidase; CEA, carcinoembryonic antigen.

**Table 6:** Characteristics of GBC patients with AJCC stage I and stage II.

Characteristic	AJCC stage I (n=37)	AJCC stage II (n=36)	P value
Imaging showed gallbladder wall thickening, n (%)	30(81.1%)	31(86.1%)	0.754
Imaging gallbladder wall thickness(*mm), median (IQR)	15(8,20)	12(10,15)	0.384
Abdominal ultrasound misdiagnosis, n (%)	17(45.9%)	16(44.4%)	1
Comprehensive preoperative imaging misdiagnosis, n (%)	9(24.3%)	13(36.1%)	0.315
Intraoperative frozen section pathology, n (%)	30(81.1%)	34(94.4%)	0.152
Intraoperative frozen section pathology misdiagnosis, n (%)	2(6.7%)	3(8.8%)	1
Unexpected gallbladder cancer, n (%)	7(18.9%)	1(2.8%)	0.152
Combined adenoma, n (%)	7(18.9%)	3(8.8%)	0.308
Combined with intraepithelial neoplasia, n (%)	10(27.0%)	6(16.7%)	0.398

AJCC, American Joint Committee on Cancer; GBC, Gallbladder carcinoma.

**Figure 7:**
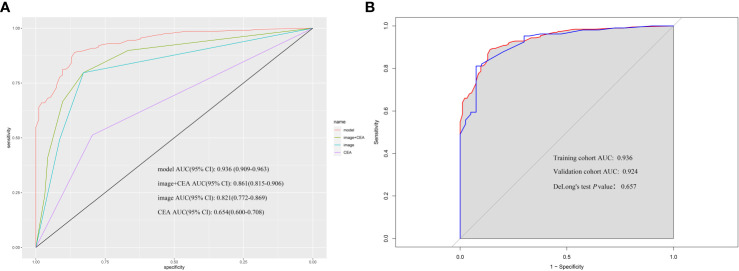
**(A)** According to nomogram scores, the performance of the novel model (nomogram), image+CEA, image, and CEA in the validation cohort. The area under the receiver operating characteristic (ROC) curve of the model, image+CEA, image, and CEA were 0.924, 0.861, 0.829, and 0.623, respectively. **(B)** The area under the ROC curve of the model in the training cohort and validation cohort was 0.936 and 0.924. The *P*-value of DeLong’s test was 0.657.

The nomogram was validated externally in one validation cohort. The AUC value of the nomogram in The Affiliated Lihuili Hospital of Ningbo University was 0.924 (**Figure 7**). The ROC curve of the nomogram based on the validation cohort was not significantly different from the training cohort (P = 0.657).

**Figure 8:**
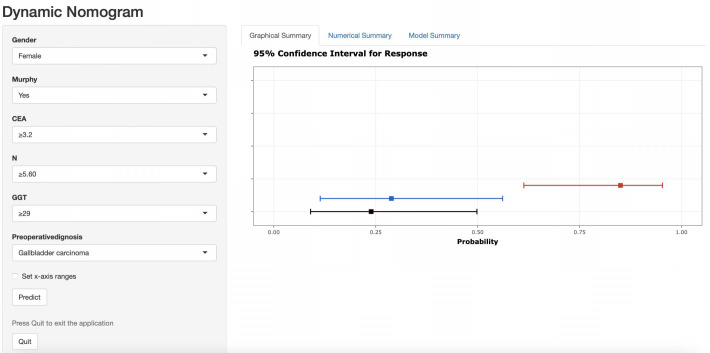
As shown in the figure above, the appropriate option was selected according to the patient's gender, Murphy's sign, CEA value, absolute neutrophil count, GGT value, and comprehensive imaging diagnosis. The right image shows the probability that the patient is diagnosed with gallbladder carcinoma.

The nomogram was then incorporated into a web page (https://nomomodel.shinyapps.io/dynnomapp/). When distinguishing between XGC and GBC, the probability of XGC could be determined after inputting the relevant data (**Figure 8**).

## Discussion

XGC, also referred to as fibroxanthogranulomatous cholecystitis or waxy histiocytic granuloma, is a rare type of benign cholecystitis caused by chronic inflammation in the gallbladder (1). XGCs’s etiology is elusive and is hypothesized to be caused by the rupture or ulceration of the Ro-Arsal sinus. This causes bile to penetrate the gallbladder wall and infiltrate the interstitial space, leading to an inflammatory response to phagocytize the bile. Microscopically, foam cells, multinucleated giant cells, fibrous tissue hyperplasia, and phagocytes with lipids in the cytoplasm can be visualized in sections (2, 3). The imaging and serological data, as well as the clinical symptoms of XGC and GBC, are similar. However, significant differences exist in their treatment modalities. The preferred treatment modality for XGC is cholecystectomy. When hilar invasion, intrahepatic bile duct dilatation, vascular invasion, or other potentially invasive conditions are present, surgical intervention should extend beyond the gallbladder to include the resection of adjacent affected organs (12). Due to severe fibrosis and inflammation, undissected Callot’s triangle, unclear anatomy, life-threatening hemorrhage, and major bile duct injury, the frequency of conversion to open surgery in patients with XGC is higher than that in patients with other forms of cholecystitis. The conversion rate is between 10% and 80% (13–15). However, XGC is ultimately a benign disease with aggressive characteristics, and the use of intraoperative frozen sections aids in distinguishing XGC from GBC. However, in a retrospective study, 42 of 142 XGC patients had a preoperative diagnosis of GBC. In this subset of patients, the accuracy of frozen sections was 93%, and the accuracy of macroscopic diagnosis by the surgeon was 50% (16).

This study’s primary novel findings include the identification of several potential indicators for distinguishing XGC from GBC. These indicators are gender, the presence of Murphy’s sign, absolute neutrophil count, levels of glutamyl transpeptidase, carcinoembryonic antigen levels, and comprehensive preoperative imaging diagnosis. The difference in the serum tumor marker levels between XGC and GBC remains controversial (17–19). Xiao et al. (20) found significant differences in absolute neutrophil count and CEA level in the results of preoperative laboratory tests between XGC and GBC, while there were no significant differences in the levels of AFP, CA12-5, and CA242. Moreover, Yu et al. (21) noted that the levels of tumor biomarkers are typically elevated in XGC and that CA19-9 and CA12-5 levels can increase the incidence of XGC and XGC misdiagnosis. XGC is frequently accompanied by Mirizzi syndrome and internal fistula, and CA19-9 levels were elevated in 26.09% of patients. In this study, CEA was the only serum tumor marker with a statistical difference and included in the model, and its optimal cutoff value was 3.2 µg/L. Furthermore, there was no significant difference in the level of AFP, CA19-9, CA12-5, and CA15-3 between the two groups.

Similarly, the female gender and negative Murphy’s sign were risk factors for GBC. Notably, the occurrence of GBC in women is two to six times higher than in men and progressively rises with advancing age (22). In patients with cholelithiasis, epigastric pain and a positive Murphy’s sign are most commonly associated with acute gallbladder inflammation. Conversely, most patients with GBC do not exhibit severe symptoms. However, there is no universally accepted consensus in this regard. Regarding laboratory tests, the count of neutrophils and the level of GGT act as protective factors in diagnosing GBC. These findings are consistent with the study conducted by Xiao et al. (20). Statistically significant differences in neutrophil counts were also found in this study. The authors contend that most patients with XGC present symptoms of acute cholecystitis, whereas patients with GBC may only exhibit radiographic abnormalities and present without significant gallbladder inflammation. Serum GGT level is extensively used for the diagnosis of liver and biliary tract diseases and predominantly reflects biliary tract involvement in clinical practice. Bile duct obstruction and other diseases lead to cholestasis. The increase in cell membrane permeability induces the synthesis of bile salts, resulting in elevated GGT levels, which enter the blood circulation through the injured biliary duct epithelial cells (23). In this study, the serum GGT level of XGC patients was generally greater than 29 U/L, indicating biliary tract injury. In XGC, the granuloma of the gallbladder wall compresses the bile duct, invades the surrounding liver, and even forms a fistula with the intrahepatic bile duct. The bile duct is uninjured in the early stages of GBC. Consequently, a GGT level lower than 29 U/L is considered an independent risk factor of GBC.

**Figure 9:**
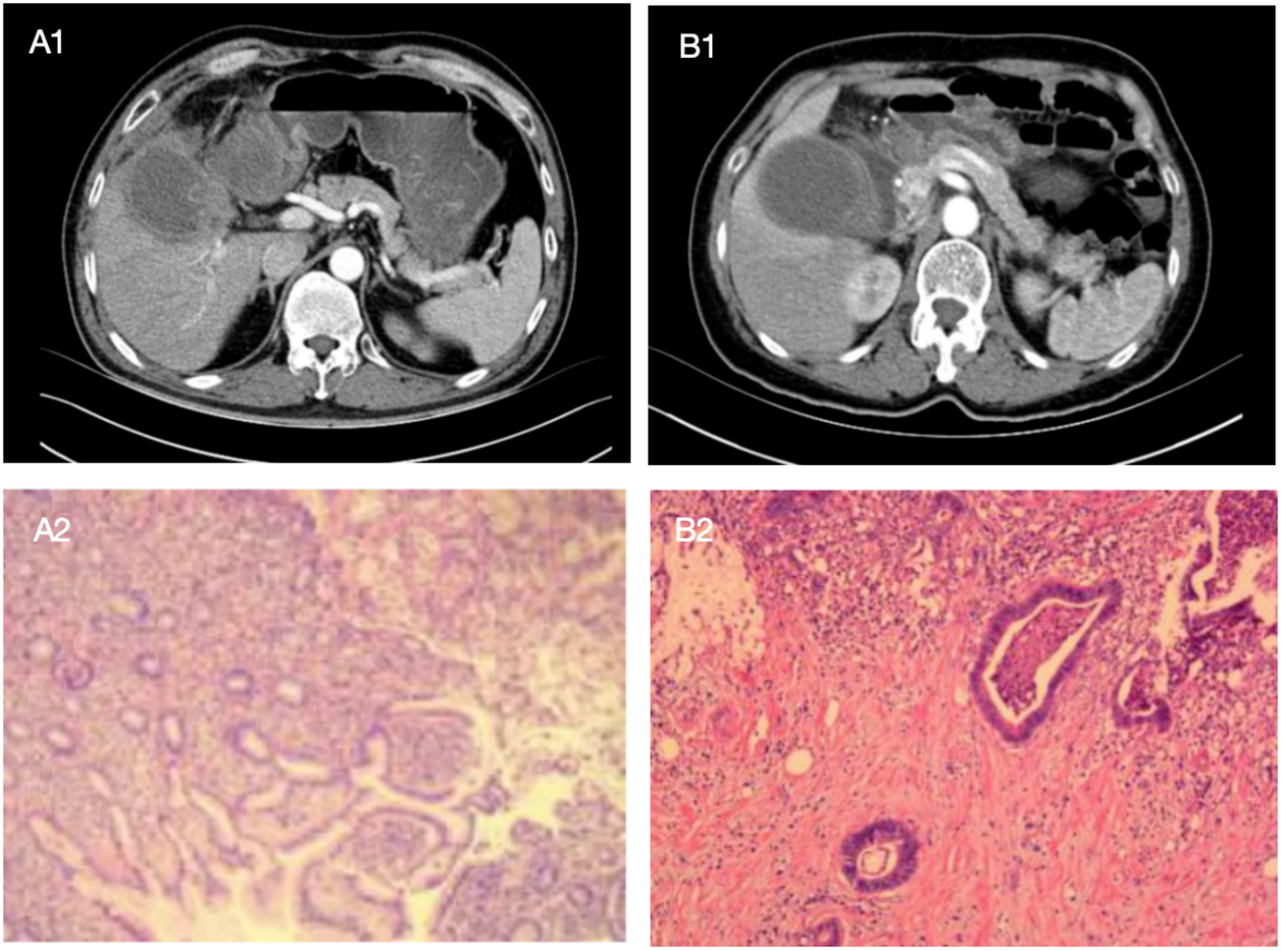
The following are two cases in which the model was applied to intraoperative decision making, with preoperative enhanced CT images of patients and postoperative pathological sections as above. Patient A showed a high-density mass with blurred margins in the arterial phase of enhanced CT. The mass was located in the neck of the gallbladder. The patient was a 52-year-old man with Murphy's sign, N 7.4*10^9/L, GGT 149U/L, and CEA 0.8ug/L. Comprehensive preoperative imaging diagnosis **(A1)** was considered suspicious of gallbladder cancer, but its probability of predicting gallbladder cancer was 0.239. In this case, the boundary and anatomical structure of the liver of the gallbladder were blurred during the operation, and the frozen section pathology showed no abnormality after complete resection of the gallbladder. The surgeon chose simple cholecystectomy based on the conjecture of the model, and no "gallbladder cancer" was found in postoperative routine pathology **(A2)**. Patient B showed a high-density mass with blurred margins in the arterial phase of enhanced CT. The mass was located in the neck of the gallbladder. The patient was a 68-year-old female with negative Murphy's sign, N 4.35*10^9/L, GGT 15U/L, and CEA 2.3ug/L. Comprehensive preoperative imaging diagnosis **(B1)** considered benign gallbladder disease, but its probability of predicting gallbladder cancer was 0.880. In this case, no gallbladder cancer was found by repeated frozen pathology during operation, but radical resection of gallbladder cancer was still performed after the surgeon conjecturing according to the model, and postoperative routine pathology **(B2)** showed "gallbladder cancer".

Similar to other biliary tract diseases, the differentiation between XGC and GBC relies on imaging modalities, including US, CT, and MRI. In XGC patients, the rupture or ulceration of the Rokitansky-Aschoff sinus facilitates bile entry into the gallbladder and infiltration into the interstitial space. However, features of the unclear hepatic silhouette or mucosal interruption are usually challenging to distinguish from GBC, with tumors invading the serous layer of the gallbladder. Interestingly, the OR for comprehensive imaging diagnosis was highest for GBC diagnosis in this study. Additionally, the complexity in differentiating XGC from GBC through imaging techniques is compounded by the thickening of the gallbladder wall, which can be a result of either acute or chronic inflammation of the gallbladder. Therefore, accurate identification of XGC and GBC by imaging technology remains challenging. Given that most patients in the study did not receive contrast-enhanced MRI, there is a lack of data regarding contrast-enhanced MRI for these patients. However, comprehensive preoperative imaging diagnosis was higher than that of enhanced CT alone in 80.7% (234/290) of cases. In XGC and GBC patients included in this study, the accuracy of preoperative diagnosis with CT enhancement and MRI enhancement was 78.2% (161/206) and 87.9% (29/33), respectively. In the comprehensive imaging diagnosis, the accuracy of XGC and GBC was 78.2% (77/93) and 78.2% (157/197). Regardless of whether a single imaging examination or comprehensive imaging modality is used, we can observe that there is a considerable proportion of GBC patients misdiagnosed as benign gallbladder diseases. Employing comprehensive imaging diagnosis can decrease the rate of such misdiagnoses in patients with GBC. Furthermore, **Figure 9** illustrates one case of XGC and one case of GBC misdiagnosed by comprehensive imaging. In these two cases, the surgeon successfully selected the appropriate surgical approach for the patient by combining intraoperative frozen pathological results and model risk assessment.

Although the AUC of enhanced CT and enhanced MR was relatively high, the differentiation between XGC and GBC remains problematic in clinical practice. First, XGC is a relatively rare disease, and most patients are diagnosed with gallbladder inflammation due to the limitations of imaging features. Only a minority of patients further undergo MR examination. Therefore, we speculate that tests with high sensitivity and specificity are difficult to popularize in this population. This could have contributed to the perplexity in differentiating the two diseases. Second, radiologists re-evaluated the diagnosis in established patients with XGC and GBC in this study. However, in clinical practice, XGC and GBC should be distinguished from other gallbladder diseases such as gallbladder polyps, gallbladder adenomyosis, and secondary GBC, while GBC in the neck of gallbladder even needs to be differentiated from diseases such as hilar cholangiocarcinoma. Therefore, the scope of diagnosis of enrolled patients was significantly limited, whereas radiologists conduct comprehensive differential diagnoses in real-life situations, which also adds to the difficulty in identifying XGC and GBC. Moreover, there was an unmet need for a preoperative diagnostic model combined with other independent risk factors to facilitate clinicians’ decision-making.

Most studies on the preoperative diagnosis of XGC and GBC focus on their differential diagnosis using various preoperative imaging examinations (10, 24, 25). However, reports on the demographics, clinical symptoms, and laboratory tests of XGC and GBC patients are scarce. This study is the first to design a diagnostic criteria chart to assist clinicians in diagnosing and treating XGC and GBC. To the best of our knowledge, this is the most extensive single-center study to construct a diagnostic nomogram for differentiating XGC from GBC. Compared with traditional methods, machine learning methods (random forest) have greatly improved the rigor of screening risk factors. By analyzing the demographics, clinical, laboratory, and imaging data of 93 patients with XGC and 197 patients with GBC, a nomogram with desirable predictive value for XGC and GBC was developed. The nomogram was subsequently well-calibrated. The valuable nomogram comprised variables such as gender, Murphy’s sign, absolute neutrophil count, glutamyl transpeptidase level, carcinoembryonic antigen level, and comprehensive imaging diagnosis. Moreover, the AUC value of the nomogram in predicting XGC and GBC was 0.936 (95% CI: 0.894–0.954). As anticipated, the nomogram outperformed any single risk factor or combination of risk factors in the predictive model (**Figures 2**, **6**). Furthermore, the data from the Affiliated Lihuili Hospital of Ningbo University were used for external validation. The results demonstrated that the model had optimal diagnostic performance (**Figure 7**).

Given that it is difficult to identify patients with XGC and GBC in clinical practice accurately, the nomogram sites were stratified into three groups based on optimal truncation points: low-, medium-, and high-risk groups. The total points, probability of GBC, sensitivity, and specificity are listed as follows: < 93, < 0.25, > 0.970, and < 0.559 in the low-risk group; 93–170, 0.25–0.75, 0.970–0.812 and 0.559–0.892 in the medium-risk group; > 170, > 0.75, < 0.812 and > 0.892 in the high-risk group. In the nomogram, the probability of the optimal cutoff value was determined to be 0.65 according to the Youden index, corresponding to 155 points. The sum of sensitivity (88.8%) and specificity (86.0%) for GBC diagnosis was the highest. Compiling comprehensive preoperative patient information and using the nomogram for scoring allows for risk stratification of GBC, offering a practical tool for clinicians. For patients in the intermediate-high risk group and those suspected of GBC based on imaging, an intraoperative frozen section is essential. When all imaging tests suggest benign gallbladder disease, but the surgeon remains unable to exclude GBC, this model aids the surgeon in deciding whether to opt for an intraoperative frozen section.

As shown in **Table 5**, patients in the training cohort were subjected to subgroup analysis according to AJCC stage of GBC, and the distribution of model characteristics of patients in each stage was compared. The proportion of women was higher than that of men in each stage. Compared with AJCC stage I, II and III patients, the proportion of Murphy’s sign positive and N ≥5.60*10^9/L increased in stage IV patients. The authors consider that this may be related to the tumor invasion of surrounding organs in patients with stage IV, which is more similar to cholecystitis in symptoms and inflammatory indicators. In addition, no significant differences were observed between stages of GGT grouping. It is worth to note that the proportion of patients with CEA ≥3.2 ug/L in stage III and IV was higher than that in stage I and II, which the authors hold that may be related to lymph node metastasis. In the preoperative comprehensive imaging diagnosis, the proportion of GBC patients with stage III and IV diagnosed as benign gallbladder diseases was only 16.7% and 10.9%.

Due to the low degree of invasion and insignificant imaging features of AJCC stage I and II, **Table 6** summarizes the imaging and pathological features of a total of 73 GBC patients in these two stages. Among the 37 patients in AJCC I stage, there were 1 case of carcinoma *in situ*, 7 cases of tumor invasion into the lamina propria, and 29 cases of tumor invasion into the muscular layer. Contrary to our expectations, the gallbladder wall thickness of stage I patients was generally higher than that of stage II patients. Although the two were not statistically significant, the authors suggest that this phenomenon may be related to a greater probability of adenoma or intraepithelial neoplasia in the former group. The missed diagnosis rate of AJCC stage I and II GBC patients by abdominal ultrasound alone was 45.9% and 44.4%, respectively, while the missed diagnosis rate of comprehensive imaging diagnosis was 24.3% and 36.1%, respectively. This suggests that abdominal ultrasound combined with cross-sectional scan is helpful to reduce the rate of missed diagnosis, and intraoperative frozen section should be performed to determine the nature of GBC when an imaging examination is considered. In these two stage GBC patients, “unsuspected gallbladder carcinoma” was found in 7 and 1 cases, respectively. Among the 64 cases of intraoperative frozen section, 2 cases were misdiagnosed, and 3 cases were only reported as intraepithelial neoplasia. Therefore, the accuracy of intraoperative frozen section in patients with AJCC stage 1 and 2 GBC in this study was 92.2%.

This study has some limitations that need to be considered. First, the nomogram was developed and validated in China, whereas XGC and GBC are uncommon conditions in Western populations. Consequently, the results of the nomogram may not be generalizable to the global population. Second, this study was retrospective, and the sample size of this study was small. Hence, the accuracy of the model must be verified by a larger sample size. Moreover, the diagnostic model constructed in this study may be unsuitable for the differentiation of benign gallbladder diseases except XGC from GBC.

Approximately 60%–70% of GBC patients are incidentally detected by pathologists after cholecystectomy for benign diseases (26, 27). In a study of 187 cases of GBC combined with 20 articles, 15 cases (8%) had normal gross appearance during the surgical operation (28). For these patients, it was difficult for surgeons to select the intraoperative frozen sections for pathological examination. Consequently, the decision to perform an intraoperative frozen section in most instances relies on the imaging examination and the judgment of the treating surgeon, indicating a lack of objective criteria for evaluation.

In conclusion, factors such as gender, Murphy’s sign, absolute neutrophil count, glutamyl transpeptidase level, serum carcinoembryonic antigen level, and comprehensive imaging diagnosis emerge as potential independent risk factors for GBC. This nomogram is anticipated to serve as a novel and precise instrument for distinguishing GBC from XGC.

## Data availability statement

The original contributions presented in the study are included in the article/supplementary material. Further inquiries can be directed to the corresponding authors.

## Ethics statement

The studies involving humans were approved by Ethics Review Committee of Zhejiang Provincial People’s Hospital. The studies were conducted in accordance with the local legislation and institutional requirements. Written informed consent for participation was not required from the participants or the participants’ legal guardians/next of kin in accordance with the national legislation and institutional requirements.

## Author contributions

TF: Conceptualization, Data curation, Formal analysis, Funding acquisition, Investigation, Methodology, Software, Writing – original draft, Writing – review & editing. YB: Data curation, Formal analysis, Funding acquisition, Validation, Writing – original draft. ZZ: Data curation, Formal analysis, Writing – original draft. ZG: Data curation, Formal analysis, Writing – original draft. TY: Data curation, Formal analysis, Writing – original draft. CZ: Supervision, Writing – original draft. HJ: Supervision, Writing – review & editing, Validation. ZX: Supervision, Writing – review & editing.
